# Evaluation of Pan-Cancer Immune Heterogeneity Based on DNA Methylation

**DOI:** 10.3390/genes16020160

**Published:** 2025-01-26

**Authors:** Yang Zhou, Jiebiao Liu, Bowen Shi, Te Ma, Peishen Yu, Ji Li, Yue Gu, Yan Zhang

**Affiliations:** 1 Faculty of Life Sciences and Medicine, School of Life Science and Technology, Harbin Institute of Technology, Harbin 150001, China; 2022112274@stu.hit.edu.cn (Y.Z.); king-domeliu@outlook.com (J.L.); 2022112278@stu.hit.edu.cn (B.S.); m602393293@gmail.com (T.M.); 18845763713@163.com (P.Y.);; 2 College of Pathology, Qiqihar Medical University, Qiqihar 161042, China

**Keywords:** DNA methylation, deconvolution algorithm, tumor immune microenvironment, clustering, pan-cancer

## Abstract

Background/Objectives: The heterogeneity of the tumor immune microenvironment is a key determinant of tumor oncogenesis. This study aims to evaluate the composition of seven immune cells across 5323 samples from 14 cancers using DNA methylation data. Methods: A deconvolution algorithm was proposed to estimate the composition of seven immune cells using 1256 immune cell population-specific methylation genes. Based on the immune infiltration features of seven immune cell fractions, 42 subtypes of 14 tumors (2–5 subtypes per tumor) were identified. Results: Significant differences in immune cells between subtypes were revealed for each cancer. The study found that the methylation values of the selected specific sites correlated with gene expression in most tumor subtypes. Immune infiltration results were integrated with phenotypic data, including survival data and tumor stages, revealing significant correlations between immune infiltration and phenotypes in some tumors. Subtypes with high proportions of CD4+ T cells, CD8+ T cells, CD56+ NK cells, CD19+ B cells, CD14+ monocytes, neutrophils, and eosinophils were identified, with subtype counts of 9, 24, 22, 13, 19, 9, and 11, respectively. Additionally, 2412 differentially expressed genes between these subtypes and normal tissues were identified. Pathway enrichment analysis revealed that these genes were mainly enriched in pathways related to drug response and chemical carcinogens. Differences in ESTIMATE scores for subtypes of seven tumors and TIDE scores for eight tumors were also observed. Conclusions: This study demonstrates the intra-tumor and inter-tumor immune heterogeneity of pan-cancer through DNA methylation analysis, providing assistance for tumor diagnosis.

## 1. Introduction

The tumor microenvironment (TME) is composed of various types of cells, extracellular matrix components, and substances such as growth factors and chemokines secreted by cells [1,2]. The heterogeneity of the TME significantly impacts tumor development and treatment [3]. Cellular heterogeneity is a primary source of tumor heterogeneity. Various cell types interact through complex dynamic processes, shaping the ecological microenvironment of the tumor [4]. CD8+ T cells, for example, exert cytotoxic effects by recognizing tumor antigens presented by MHC class I molecules and releasing IFN-γ and granzyme B [5]. In contrast, CD4+ T helper cells release IL-2, IL-4, and IL-17, which aid in the activation of other immune cells, such as macrophages and B cells [6]. Tumor-associated macrophages are also highly influential. They can be polarized into M1 macrophages, which secrete IL-1β and TNF-α to promote inflammation and anti-tumor immunity, or M2 macrophages, which produce IL-10 and TGF-β, contributing to immune suppression and tumor progression [7]. Additionally, regulatory T cells (Tregs), by secreting TGF-β and IL-10, promote immune tolerance, facilitating a tumor-friendly microenvironment [5,8]. Natural killer (NK) cells also play a pivotal role in immune surveillance, producing IFN-γ and targeting tumor cells for destruction [8]. Finally, neutrophils in the TME secrete vascular endothelial growth factor A (VEGFA) and matrix metalloproteinase 9 (MMP9), promoting tumor growth by supporting angiogenesis and invasion [9]. These interactions influence the tumor’s phenotype, characteristics, and progression, impacting tumor invasion, metastasis, and drug response, ultimately affecting patient outcomes. These factors play a crucial role in tumor initiation and progression [10]. Therefore, studying cellular heterogeneity within the tumor microenvironment is critical for understanding the mechanisms of tumor development, identifying new therapeutic targets, and developing personalized treatment strategies.

Immune cells play a key role in the tumor microenvironment by interacting with tumor cells and participating in processes such as immune surveillance, immune evasion, and immune editing [11,12]. The degree and type of immune cell infiltration can significantly influence tumor development and response to treatment, making immune cells important therapeutic targets in cancer treatment [13]. Therefore, understanding the role of immune cells in the tumor microenvironment is crucial for developing new therapeutic strategies and predicting treatment responses. Against this backdrop, studies have been conducted to assess the heterogeneity of the tumor immune microenvironment.

As a complex disease, molecular alterations in tumors are a major source of their heterogeneity [14]. DNA methylation, an important epigenetic modification and one of the key molecular features, can influence gene expression without altering the DNA sequence. In the tumor microenvironment, the DNA methylation patterns of immune cells can reflect their differentiation status and functions [15], thereby correlating with the heterogeneity of the tumor microenvironment and the extent of immune infiltration. By analyzing specific DNA methylation sites, the composition of immune cells within the tumor microenvironment can be predicted, helping to uncover the complexity of the tumor immune microenvironment. Previous studies on immune infiltration in the tumor microenvironment have primarily evaluated its heterogeneity by combining techniques such as flow cytometry or gene expression profiling [16]. These studies typically focus on specific tumor types. In contrast, this research provides a novel perspective by utilizing DNA methylation molecular features to explore this process, examining it at a pan-cancer level to identify common tumor characteristics. Existing studies have shown that the primary determinant of the epigenome is the cell type-specific differentiation process, rather than genetic differences or environmental factors [17]. In brief, the DNA methylation patterns of the same cell type are relatively conserved across different samples. Building on this research foundation, it is possible to use the methylation data profiles of known immune cell types to deconvolute tissue samples, thereby assessing the infiltration of corresponding immune cell types within tumor tissues.

In this study, we employed a Shannon entropy-based method to identify 1256 specific DNA methylation sites associated with seven immune cell types. These sites were used to deconvolute a total of 5323 tumor samples from 14 different cancer types in the TCGA database, allowing for the evaluation of immune infiltration fractions within the corresponding tumor microenvironment. Differential subtypes in 14 cancers were divided based on the immune infiltration fractions, which were closely associated with patient survival, clinical stage, and the expression of immune-related genes. This demonstrates the accuracy and reliability of immune infiltration-based classification for pan-cancer analysis, advancing progress in the field of cancer diagnosis.

## 2. Materials and Methods

### 2.1. Data Collection

DNA methylation profiles, gene expression profiles, and clinical phenotypic data for the tissues used in this study come from The Cancer Genome Atlas (TCGA, https://portal.gdc.cancer.gov) (accessed on 28 August 2024), which includes 5323 tumor samples and 700 normal samples from 14 tumor types, including breast invasive carcinoma (BRCA), cholangiocarcinoma (CHOL), colon adenocarcinoma (COAD), esophageal carcinoma (ESCA), head and neck squamous cell carcinoma (HNSC), kidney renal clear cell carcinoma (KIRC), kidney renal papillary cell carcinoma (KIRP), liver hepatocellular carcinoma (LIHC), lung adenocarcinoma (LUAD), lung squamous cell carcinoma (LUSC), pancreatic adenocarcinoma (PAAD), prostate adenocarcinoma (PRAD), thyroid carcinoma (THCA), and uterine corpus endometrial carcinoma (UCEC). The DNA methylation profiles for the seven immune cell types used in this study come from the Gene Expression Omnibus (GEO, https://ncbi.nlm.nih.gov/geo) (accessed on 3 August 2012) database [18], under GSE35069, including CD4+ T cells (CD4), CD8+ T cells (CD8), CD56+ NK cells (CD56), CD19+ B cells (CD19), CD14+ monocytes (CD14), neutrophils (Neu), and eosinophils (Eos). The DNA methylation profiles used in this study were generated by the Illumina Human Methylation 450K platform (illumina, San Diego, USA). The data distributions are shown in Table 1.

### 2.2. Cell Type-Specific DNA Methylation Genes Selection

To facilitate subsequent analysis, the collected methylation data were compared with the chip platform files. The chip probe locations were mapped to specific gene sites, and the methylation values corresponding to the same gene site were averaged and presented in the data table. After preprocessing, the study initially identified 20,000 gene sites. Since the methylation values for the seven immune cell types are relatively conserved in most genes, these genes contain minimal information and are considered noise. Before constructing the deconvolution model, the study used a Shannon entropy-based method to preliminarily filter out a subset of significantly specific gene sites, thereby removing these noisy data points.

Quantitative Differentially Methylated Regions (QDMR) was used to identify specific methylation sites [19]. This software is designed to select features based on the Shannon entropy method. QDMR software (version 1.0) quantifies methylation differences using Shannon entropy [20]. Shannon entropy is a method for quantifying differences and uncertainty within a dataset, and the formula is as follows:

(1) $$H_{0}=-\sum_{s=1}^{N}p_{s/r}\log_{2}\left(p_{s/r}\right)$$

Here, *H*_0_ represents Shannon entropy, *N* represents the number of samples, and *p_s/r_* is the relative methylation level of each sample in a specific region, reflecting the proportion of the methylation status of that sample in the region relative to the total methylation level across all samples. This study inputs the downloaded and preprocessed DNA methylation data into the QDMR software and uses the Shannon entropy formula to calculate the Shannon entropy value for each gene site with respect to the 7 immune cell types. A higher Shannon entropy value indicates that the gene site contains more information, i.e., it is more specific and exhibits greater significance. The threshold used in the study was determined based on a methylation probability model, TSNE, which controls the extent of random biological variation between samples. This method quantifies DNA methylation differences to select the immune cell type-specific methylation genes for the 7 immune cell populations. These specific gene sets are then used as input genes for the downstream deconvolution algorithm to filter out noise, thereby enhancing the accuracy and computational efficiency of the model.

### 2.3. Pan-Cancer Tissue Deconvolution

Convolution is a mathematical operation that describes how one function (such as a signal or image) interacts with another. It involves sliding a “filter” or “kernel” (usually a small matrix) across the input and performing element-wise multiplication, followed by summing the results to produce an output. Deconvolution is the inverse process of convolution and is widely used in signal and image processing. The DNA methylation data in tissues were obtained from the convolution of cell type-specific methylation data and the proportions of different cell types [16,21]. This project utilizes a deconvolution algorithm to calculate the cell subtypes and their corresponding proportions in the tissue based on the DNA methylation characteristic matrix of cancer tissues. Let *y_i_* represent the methylation value of the corresponding gene, where *i* = 1, 2, …, *i*, and assume the methylation level *x* = *x_ij_* for gene *i* and deconvolution cell type *j* = 1, 2, …, *j*. For each evaluated sample, the training process of the linear regression model is as follows:

(2) $$y_{i}=\sum_{j=1}^{j}a_{i}x_{ij}+\varepsilon_{i}$$

Here, *a*_1_ to *a_j_* represent the infiltration fractions of the immune cells in the tissue, and *ε*_1_ to *ε_i_* represent the random errors.

We performed quadratic programming (QP) fitting of the linear model and generated the following constraints [22,23]:

(3) $$\min\sum_{i=1}^{i}\varepsilon_{i}^{2}$$

Here, *ε*_1_ to *ε_i_* represent the random errors.

(4) $$\sum_{j=1}^{j}\alpha_{j}\leq 1,\;0<\alpha_{j}<1$$

Here, *α*_1_ to *α_j_* represent the infiltration fractions of the immune cells in the tissue.

The quadratic programming in this study was implemented using the R package “quadprog” (version 1.5-8) and an immune cell infiltration value is recorded only when it exceeds a certain threshold (set to 0.01 in this study). For a given tissue, the sum of the infiltration values of the 7 immune cell types must be ≤1, and the infiltration fraction of any individual immune cell type must be positive.

As mentioned earlier, this study used a cell type methylation matrix in this model, which incorporated the methylation data of 1256 specific gene sites for 7 immune cell types. Additionally, the methylation values of these 1256 sites across 6023 tissues were selected to form the tissue methylation characteristic matrix. The methylation characteristic matrix for each tissue and the methylation matrix for the 7 cell types were input into the model. The model was optimized iteratively through the constraint results, adjusting the model’s tuning parameter b and other noise terms, and repeatedly refining the model conditions to achieve the best fit. Finally, the model output the immune infiltration fractions for the 7 immune cell types across the 6023 samples.

After generating the immune infiltration fractions results for 5323 tumor samples and 700 normal samples, the study used statistical analysis methods to evaluate the significance of the differences in immune infiltration fractions between tumor samples and their corresponding normal samples for the 7 immune cell types. Furthermore, variance analysis was applied to assess the significance of differences in immune infiltration fractions across 14 cancer types.

### 2.4. Clustering Based on Immune Infiltration

After outputting the immune infiltration results, clustering is performed. The clustering method used in this study is the K-means algorithm. K-means clustering is an iterative unsupervised learning algorithm widely used for partitioning a dataset into k distinct clusters based on feature similarity. The method seeks to minimize the intra-cluster variance while maximizing inter-cluster separation. Each cluster is represented by its centroid, defined as the mean position of all the points in the cluster.

Formally, given a dataset *X* = {*X*_1_, *X*_2_, …, *X_n_*} with n data points in a d-dimensional space and a predefined number of clusters *k*, the algorithm aims to partition the data into *k* clusters *C* = {*C*_1_, *C*_2_, …, *C_k_*}. The objective function to be minimized is the sum of squared Euclidean distances between each data point and its corresponding cluster centroid,

(5) $$J=\sum_{i=1}^{k}{\sum_{x\in C_{i}}\left||x-\mu_{i}|\right|}^{2}$$

where *μ_i_* is the centroid of cluster *C_i_*, calculated as,

(6) $$\mu_{i}=\frac{1}{\left|C_{i}\right|}\sum_{x\in C_{i}}x$$

By minimizing intra-cluster variance, k-means ensures compact and well-separated clusters, which are crucial for biological data analyses, such as identifying cancer subtypes or immune cell infiltration patterns. The clustering process in this study was implemented in R using the “ConsensusClusterPlus” package [24] (version 1.70.0), applying the k-means clustering algorithm based on Euclidean squared distance to divide each subsample into 2–20 groups. This process was repeated 100 times, and the stability of each cluster was determined using the empirical cumulative distribution function (CDF). The principle of the CDF method involves calculating the area under the CDF curve for different numbers of clusters. This curve represents the cumulative distribution of consensus indices for all pairs of items across multiple iterations of clustering. The consensus index for a pair of items indicates the proportion of times the items are grouped together across iterations. As the number of clusters increases, the area under the CDF curve initially grows, reflecting improved stability. The optimal number of clusters is identified at the point where the increase in the area becomes relatively stable or exhibits diminishing returns. This point indicates that adding more clusters does not significantly enhance stability, ensuring a balance between interpretability and robustness. This approach helps avoid over-clustering, which can lead to fragmentation, and under-clustering, which can oversimplify the data structure. This study selected the minimum number of clusters where the area under the CDF curve showed relatively constant change, indicating maximum stability. The most appropriate number of divisions was used to perform subgrouping, and the clustering results were output.

After outputting the clustering results, the study uses the t-SNE dimensionality reduction method to visualize the immune cell infiltration proportion features of the 7 immune cell types in the samples [25]. This allows exploration of the significance of immune infiltration differences between different subtypes of the 14 cancer types and evaluates the clustering effectiveness. Additionally, the study compares the immune infiltration with that in normal tissues and annotates each cluster obtained from the clustering. Statistical tests should be conducted for each cluster and normal tissue under each immune cell condition to determine significant increasing or decreasing trends, making the results statistically reliable for annotation. After annotation, downstream analysis can be performed based on subtypes. Finally, the study obtains annotated results for each cancer type based on immune infiltration clustering and the immune infiltration differences between each subtype and normal tissues, providing feasible suggestions for clinical diagnosis [26]. Furthermore, the study combines gene expression data to evaluate the correlation between the methylation values of the selected specific gene sites and gene expression values in each subtype of the tumor, providing data support for downstream analyses based on gene expression data.

### 2.5. Analysis Based on Phenotypic Data

Phenotypic data are the most closely related to clinical treatment. After analyzing the corresponding molecular targets, we need to combine them with phenotypic data in order to provide assistance for clinical treatment. Since this study is conducted at the pan-cancer level, the phenotypic data to be used must be universal, meaning they should be present at the cancer type level for every cancer. Phenotypic data that exist only for certain cancers and molecular subtype data do not meet the requirements for this study. Based on the above conditions, the study downloaded the clinical data of 14 types of cancers from the TCGA (https://portal.gdc.cancer.gov) (accessed on 28 August 2024) database, filtered and denoised the data, and removed phenotype data with numerous missing values. The survival data, clinical stage, and tumor size data were then selected as ideal targets for downstream analysis. Survival data are obviously some of the essential phenotypic data for any cancer, with universal significance. In addition to survival data, clinical stage and tumor size are also relatively universal data, and their data volume in the clinical information of 14 cancer types is comprehensive, making them ideal clinical observation indicators. Kaplan–Meier plots were used to illustrate overall survival among the subtypes as defined by immune infiltration fractions and to test whether the subtypes differed significantly in survival, thus providing help for clinical cancer diagnosis and prognosis [27]. The significance of differences in survival between the subtypes was estimated using the log-rank test. Survival analysis was conducted using the R package, “survival” (version 3.6-4).

### 2.6. Analysis Based on Transcriptome

The above study is based at the cellular level. Whether predicting clinical treatment targets or patient prognosis, it often requires precision at the molecular level. The final clinical recommendations should also be implemented at the molecular level, providing highly credible molecular targets for drug development and clinical treatment references [28]. Therefore, this study downloaded gene expression profile data for 5323 tumor samples and 700 normal samples from the TCGA (https://portal.gdc.cancer.gov) (accessed on 28 August 2024) database. After obtaining the 7 immune cell infiltration results for 14 cancer types and dividing them into different subtypes, differentially expressed genes analysis was performed between the increased subtypes of the 7 immune cells and the corresponding normal tissues in the 62 subtypes of 14 cancers. The differential gene sets of the 7 immune cells, which were strictly selected, were subjected to pathway enrichment, and gene sets and metabolic pathways with higher significance were identified. Simultaneously, the study also took the union of the differential gene sets of the 7 immune cells to obtain a comprehensive differential gene set. Pathway enrichment was performed, observing the relationship between metabolic pathways with higher significance, immune infiltration, and tumors for reference in clinical drug development and treatment [29].

In addition, this study also conducted immune checkpoint evaluation, ESTIMATE scoring, and TIDE scoring based on gene expression data from 5323 samples. The immune checkpoint selected in this study is based on the immune checkpoint genes (ICGs) published in previous research [30]. This gene set contains 79 genes and is a widely used immune checkpoint gene set. ICGs play a critical role in regulating immune responses, primarily by suppressing or activating immune cell activity to maintain immune system balance. These genes, including CTLA-4, PD-1, and PD-L1, are key players in immune co-stimulatory and co-inhibitory signaling pathways. In the tumor microenvironment, tumor cells often evade immune surveillance by activating immune checkpoint pathways, thereby suppressing the immune system’s recognition and attack mechanisms. Therefore, ICGs are critical targets for immune checkpoint blockade (ICB) therapy. By inhibiting the function of these genes, the immune system’s ability to attack tumors can be restored, exerting anticancer effects. This study displayed the differential significance of the expression of these 79 genes in the subtypes of 14 cancers, assessing the differentially expressed genes in immune checkpoint genes between cancer subtypes.

ESTIMATE scoring is a method that evaluates immune infiltration score [31], stroma infiltration score, and tumor purity in the tumor microenvironment based on gene expression data. This method uses preselected stromal- and immune-related gene sets and employs single-sample gene set enrichment analysis (ssGSEA) to calculate stromal scores, immune scores, and the composite ESTIMATE score for each sample.

Tumor Immune Dysfunction and Exclusion (TIDE) is an algorithm for predicting cancer patients’ responses to immunotherapy by analyzing T-cell dysfunction and exclusion mechanisms in the tumor microenvironment [32]. This method categorizes tumors into high-infiltration and low-infiltration groups based on the infiltration fractions of cytotoxic T lymphocytes (CTLs). For high-infiltration tumors, TIDE predicts non-responding patients by analyzing features of T-cell dysfunction; for low-infiltration tumors, it predicts responses by examining features of T-cell exclusion. By calculating the correlation between tumor expression profiles and relevant features, TIDE generates dysfunction and exclusion scores, which are combined into a TIDE score to assess patients’ potential response to immunotherapy. A high TIDE score generally indicates that a patient is unlikely to respond to immunotherapy, while a low TIDE score suggests potential benefit from the treatment [33]. The study scored 5323 tumor samples based on these two scoring methods and displayed the results, assessing whether there are significant differences in scores between different tumor subtypes. This further validates the accuracy and credibility of the tumor subtyping based on immune infiltration and proves the accuracy of the conclusions drawn from the study.

## 3. Results

### 3.1. Overview of the Study

**Figure 1:**
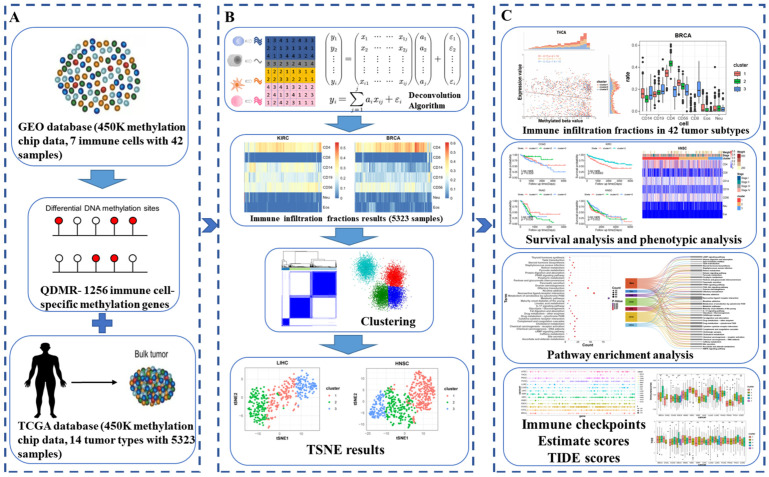
Overview of the study. (**A**) Data collection and the identification of immune cell-specific methylation genes. (**B**) Development of a deconvolution algorithm to assess the immune infiltration fractions in pan-cancer. (**C**) Multi-omics integrative analysis.

**Figure 2:**
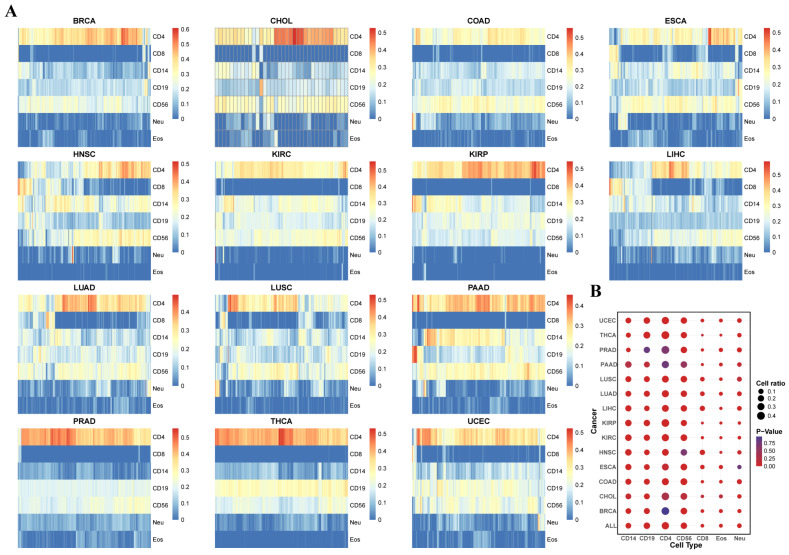
The immune cell infiltration fractions in 14 tumors. (**A**) The results of the immune cell infiltration fractions in 14 tumors. (**B**) The differential immune cell infiltration fractions between tumor samples and normal samples in every tumor.

To investigate DNA methylation as a molecular marker for assessing immune heterogeneity in pan-cancer, this study utilized 42 samples from seven immune cell types in the GEO database. A total of 1256 immune cell-specific methylated genes (see Materials and Methods) were identified by using the QDMR software in Figure 1A. DNA methylation profiles of 5323 samples across 14 cancer types obtained from the TCGA database were integrated to calculate the infiltration proportions of seven immune cell types for each of the 14 cancers based on the deconvolution algorithm in Figure 2B. To further evaluate immune heterogeneity in the tumor microenvironment, we performed clustering based on the infiltration fractions of the seven immune cells, identifying immune subtypes for the 14 cancers. By analyzing the immune infiltration fractions, survival outcomes, pathway enrichment, and immune scores of different immune subtypes across the 14 cancers, we demonstrated the differences mediated by DNA methylation among these subtypes.

### 3.2. Evaluation of Tumor Microenvironment Immune Infiltration Based on Immune Cell Type-Specific Genes

A total of 1256 specific methylated genes by the QDMR with the number of specific genes corresponding to each of the seven immune cell types were shown in the Supplementary Materials (Table S1). These specific genes were subjected to pathway enrichment analysis, and statistically significant pathways were displayed (Figure S1, Table S2). From the results, it is visually clear that most of the pathways enriched with the selected specific gene clusters are related to the immune system. This indirectly supports the accuracy of the specific gene set used for deconvolution training in this project and provides a more precise data foundation for subsequent downstream analysis.

The immune infiltration fractions of seven immune cell types in 5323 samples from 14 cancers were evaluated through the 1256 specific genes (Figure 2A and Figure S2). The immune infiltration reveals that CD4+ T cells generally show higher infiltration levels across the 14 cancer types. The average immune infiltration fractions of the CD4+ cells for 14 tumors is 0.3274. Unlike other cancers, the fraction of CD4+ T cell immune infiltration in head and neck squamous cell carcinoma, liver cancer, lung adenocarcinoma, and lung squamous cell carcinoma was significantly decreased compared with other cancers. In contrast, the fraction of CD8+ T cell immune infiltration was significantly increased in these cancers, which could serve as a distinguishing feature of these cancer types. The average immune infiltration fractions of the CD8+ cells for 14 tumors is 0.0339.

After conducting statistical tests on the immune infiltration fractions between each type of cancer and its corresponding normal tissue (Figure 2B), it was observed that most immune cell infiltration fractions showed significant differences between tumor and normal patients. Specifically, the fractions of CD4+ T cell and CD8+ T cell infiltration showed consistent increasing and decreasing trends in 14 cancer types compared with normal tissues. Compared with normal tissues, CD8+ T cell infiltration was significantly increased in most cancer types, while CD4+ T cell infiltration was significantly decreased in most cancers. Furthermore, the immune cell infiltration trends in most tumor types showed a clear upward or downward preference. That is to say, tumors with increased invasiveness are significantly better than tumors with decreased invasiveness, and vice versa. Based on the *t*-test results of cancer patients and normal tissues, it was clearly shown that at least 9 of the 14 cancer types had significant differences in the immune infiltration fractions of seven immune cells between cancer patients and normal tissues (Figure 2B). This suggests that immune infiltration is a significant marker for distinguishing cancer patients from normal patients, indirectly proving the feasibility and practical value of this study. Furthermore, the results of the ANOVA show that the immune infiltration fractions of the seven immune cell types significantly differ across the 14 cancer types. Therefore, the immune infiltration fractions derived from DNA methylation values can serve as one of the signature features of the 14 cancer types, further supporting the significance and value of our study.

### 3.3. The Subtypes with Immune Infiltration Characteristics in 14 Tumors

**Figure 3:**
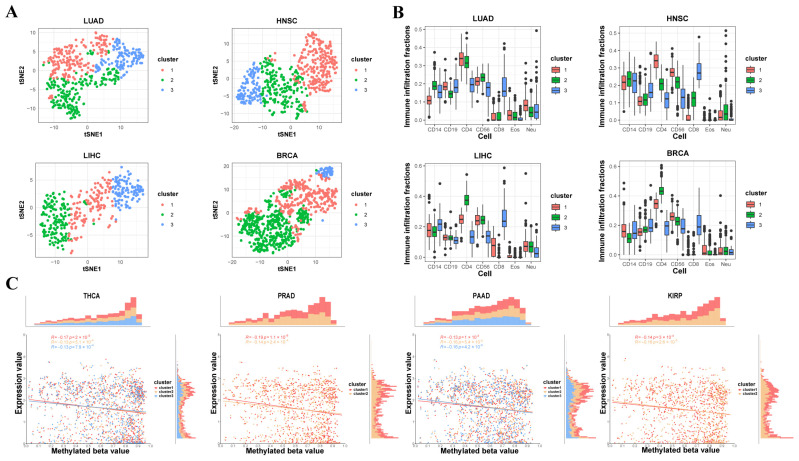
The immune cell infiltration fractions in tumor subtypes. The “cluster” in the legend represents the immune subtypes of the 14 cancers by clustering. (**A**) TSNE showed subtypes in lung adenocarcinoma, head and neck squamous cell carcinoma, liver hepatocellular carcinoma, and breast invasive carcinoma. (**B**) Comparison of the immune cell infiltration fractions between lung adenocarcinoma, head and neck squamous cell carcinoma, liver hepatocellular carcinoma, and breast invasive carcinoma subtypes. (**C**) The correlation between DNA methylation and gene expression of the 1256 sites in thyroid carcinoma, prostate adenocarcinoma, pancreatic adenocarcinoma, and kidney renal papillary cell carcinoma subtypes.

To more accurately classify the tumor tissues into different subtypes based on immune infiltration fractions, the study performed clustering and dimensionality reduction on the seven immune cell infiltration fractions from 5323 samples of 14 tumors. In the study, t-SNE was applied to reduce the dimensionality of immune cell infiltration data across multiple tumor types, enabling the visualization of distinct distributions of immune cell fractions within each tumor subtype. t-SNE (t-distributed Stochastic Neighbor Embedding) is a machine learning technique primarily used for dimensionality reduction, often applied to visualize high-dimensional data in a lower-dimensional space (typically 2D or 3D) for easier interpretation. Based on the t-SNE results, it is evident that within each tumor type, the distributions of the seven immune cell infiltration fractions after dimensionality reduction show significant differences, confirming the good clustering performance and providing data support for subsequent analysis based on the clustering results (Figure 3A and Figure S3). The TSNE results of lung adenocarcinoma, head and neck squamous cell carcinoma, liver hepatocellular carcinoma, and breast invasive carcinoma are shown (Figure 3A) and the results of the other 10 tumors are shown in the Supplementary Materials (Figure S3).

This study revealed differences in seven immune cell infiltration results among different subtypes based on the clustering of 14 tumor types (Figure 3B and Figure S4). Based on the clustering results, it is evident that the immune infiltration fraction of CD4+ T cells shows large variation across almost all subtypes of the tumors. As mentioned earlier, due to the near-zero values of CD8+ T cells and Eos cell immune infiltration in the overall tumors, the infiltration fractions of these two cell types do not show much variation between tumor subtypes. For the other four types of cells, different degrees of variation are observed between subtypes of different tumor types. However, for most tumors, the immune infiltration of these cell types still shows significant differences, which can be verified in subsequent statistical tests. From the perspective of specific tumor types, the immune infiltration differences between subtypes of squamous lung cancer and breast cancer are more significant, while the immune infiltration between subtypes of cholangiocarcinoma and renal papillary cell carcinoma is more conservative, which is also confirmed in subsequent statistical tests. The clustering results of lung adenocarcinoma, head and neck squamous cell carcinoma, liver hepatocellular carcinoma, and breast invasive carcinoma are shown (Figure 3B) and the results of the other 10 tumors are shown in the Supplementary Materials (Figure S4).

Next, the study combines the results of immune infiltration fractions of subtypes after clustering and uses the corrected *t*-test to evaluate the increase or decrease of each immune cell proportion in each cancer type compared to its corresponding normal tissue. As a result, each subtype annotation has seven dimensions, each with three possibilities: increase, decrease, or no change. The annotation results of the 14 cancer types based on the above information, along with the summarized annotation results by cell type, are presented in the Supplementary Materials (Table S3).

From the perspective of the 14 tumors as a whole, the data show that the immune infiltration differences between the seven cell types across subtypes of most tumors are significant. Even for the relatively conservative cholangiocarcinoma, each of the three subtypes has significant immune infiltration features, allowing classification based on immune infiltration. For the more significantly divergent squamous lung cancer, except for one subtype where CD56+ cell infiltration does not show significant differences compared to the normal tissue, each of the other subtypes exhibits significant differences at the cell type level compared to the normal tissue. This confirms that immune infiltration plays a crucial role in classifying tumor subtypes. From the perspective of the seven immune cell types, among the 42 subtypes, CD4+ T and CD14+ cell infiltration fractions show significant differences between 35 subtypes and their corresponding normal tissues. Even for the relatively less divergent CD8+ T cells and Eos cells, their infiltration fractions still show significant differences in 27 and 26 subtypes, respectively, compared to the normal tissue. From this perspective, it further confirms the preliminary conclusions derived from the clustering results and the feasibility of classifying tumors based on immune infiltration fractions.

As shown clearly in the summary table, the reduction in Neu cell infiltration fraction is the greatest, accounting for more than half of all samples in the study. Additionally, significant features include the increase in CD56+ cells, CD14+ cells, and CD8+ cells, as well as the decrease in CD4+ and CD19+ cells. These findings align with the preliminary features obtained through the clustering results earlier, providing mutual verification of the results, proving the accuracy of the study, and offering highly credible targets for clinical drug development and treatment strategies, with practical significance.

Furthermore, to provide data support for the subsequent research based on gene expression data, this study also combined gene expression data to assess the correlation between the methylation values and expression values of the specific sites selected in the 62 tumor subtypes. The results for thyroid carcinoma, prostate adenocarcinoma, pancreatic adenocarcinoma, and kidney renal papillary cell carcinoma are shown in the figure below and the results of the other 10 tumors are in the Figure 3C and Supplementary Materials (Figure S5). By comparing with the random correlation results, the study found that the correlation between methylation values and gene expression values was significantly higher than the random correlation coefficient in nearly all tumor subtypes, proving that the methylation values of the specific sites selected in the study have a certain correlation with gene expression values in the 62 tumor subtypes.

### 3.4. The Different Subtypes Showed the Differential Phenotypes

**Figure 4:**
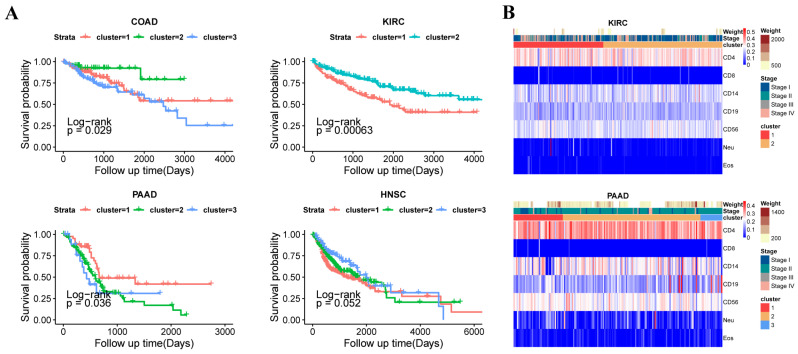
The phenotypic characteristics in tumor subtypes. The “cluster” in the legend represents the immune subtypes of the 14 cancers by clustering. (**A**) The survival analysis of the subtypes in colon adenocarcinoma, kidney renal clear cell carcinoma, pancreatic adenocarcinoma, and head and neck squamous cell carcinoma. (**B**) Comparison of the phenotypic characteristics and the immune cell infiltration fractions between subtypes in kidney renal clear cell carcinoma and pancreatic adenocarcinoma subtypes.

To investigate the association between immune infiltration scores derived from DNA methylation assessment and clinical phenotypes, and to evaluate tumor prognosis, this study explored the relationship between immune infiltration and phenotypes. The relevant results are shown in the figures (Figure 4A,B).

From the survival analysis of 14 types of cancer, it can be seen that there are significant differences between the survival of each subtype, suggesting a substantial correlation between immune infiltration fractions and patient survival (Figure 4A and Figure S6). For specific tumors, using *p* < 0.05 as the threshold, a significant correlation between survival and the immune infiltration-based subtypes is observed in Kidney Renal Clear Cell Carcinoma (KIRC), Colorectal Adenocarcinoma (COAD), Pancreatic Adenocarcinoma (PAAD), and Head and Neck Squamous Cell Carcinoma (HNSC). Among them, KIRC shows the highest correlation, with a confidence probability (*p*-value) of 0.00063. It can be clearly observed that in the two subtypes of KIRC, cluster 2 shows significantly better survival than cluster 1 (Figure 4A). Based on the results of the seven immune cell immune filtration fractions above, cluster 2 shows a significant decrease in CD14+ and Neu cell immune filtration fractions, and a significant increase in CD4+ T cell infiltration (Figure 3B and Figure 4A). In COAD, cluster 2 shows significantly better survival than the other subtypes. Based on the results of the seven immune cell immune filtration fraction, cluster 2 shows a significant decrease in CD14+ cell infiltration and a significant increase in CD4+ T and Neu cell immune filtration fractions. In PAAD, cluster 1 shows significantly better survival than the other subtypes and a significant decrease in CD14+ cell infiltration as well as a significant increase in CD19+ cell infiltration. In HNSC, cluster 3 shows significantly better survival than the other subtypes and a significant increase in CD8+ and CD19+ cell immune filtration fractions (Figure 3B and Figure 4A). Based on the above information, it can be inferred that the decrease of CD14+ cell infiltration and the increase of CD19+ cell infiltration are among the hallmark features of better survival. Additionally, significant differences in survival are also observed between subtypes in ESCA and LUAD, further confirming that the subtypes defined by immune infiltration fractions are significantly correlated with survival data, and immune infiltration fractions can predict survival to some extent. Here, we present the survival results of the mentioned above four types of tumors in the main text (Figure 4A). The survival results of other tumors are shown in the Supplementary Materials (Figure S6).

Based on the annotation of immune infiltration fractions for seven immune cell types across 14 tumor types, the results show that the five clinical stages are well classified in most tumors (Figure 4B and Figure S7). However, in some tumor subtypes, the correlation between immune infiltration and clinical stage or tumor size is relatively low, with higher noise. From the results, it can generally be observed that the decrease of CD4+ T cell infiltration is often associated with early-stage tumors. As CD4+ T cell immune infiltration increases, the tumor typically progresses to later stages, with tumor size also increasing. This corresponds with the results of the survival curve, further validating the accuracy of the study’s conclusions, and providing reliable features and strategies for clinical prognosis. The results of the kidney renal clear cell carcinoma and pancreatic adenocarcinoma are in the main text (Figure 4B). The survival results of other tumors are shown in the Supplementary Materials (Figure S7).

### 3.5. The Analysis of Transcriptome Between Subtypes

**Figure 5:**
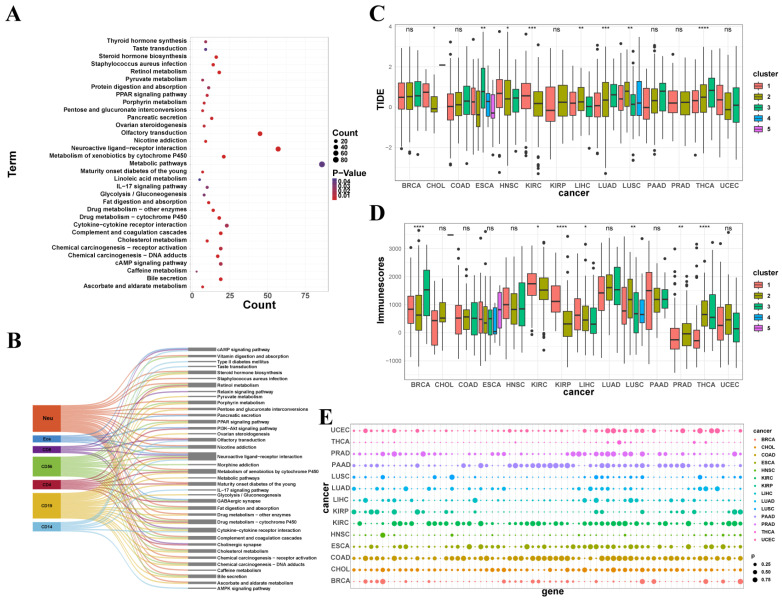
The analysis based on the transcriptome. (**A**,**B**) KEGG pathway of differential gene enrichment between subtypes with high proportions of seven immune cell types and normal samples. (**C**) The TIDE scores of 42 subtypes in 14 tumors. The meanings of the symbols in the figure are listed below: * indicates statistical significance with *p* ≤ 0.05, ** with *p* ≤ 0.01, *** with *p* ≤ 0.001, **** with *p* ≤ 0.0001, and ns indicates no statistical significance (*p* > 0.05). (**D**) The ESTIMATE scores of 42 subtypes in 14 tumors. The meanings of the symbols in the figure are listed below: * indicates statistical significance with *p* ≤ 0.05, ** with *p* ≤ 0.01, **** with *p* ≤ 0.0001, and ns indicates no statistical significance (*p* > 0.05). (**E**) The analysis of the immune checkpoint genes of 42 subtypes in 14 tumors. The “cluster” in the legend represents the immune subtypes of the 14 cancers by clustering.

In order to provide precise molecular targets for clinical cancer treatment, the study evaluated the differentially expressed genes between subtypes of high proportions of seven immune cell types and normal samples, followed by pathway enrichment. The pathway enrichment results are shown (Figure 5A,B and Figure S8). Based on the comparison of the seven immune cell differential gene sets, it clearly shows that each of the seven immune cell types has more than 200 differential genes, with CD19+ immune cells and Neu cells having relatively more differential genes, 619 and 448, respectively. The smallest number of differential genes is found in CD4+ cells, with 214 genes. This indicates that gene expression is one of the important factors influencing the different fractions of tumor immune infiltration. To investigate which pathways these genes influence in immune infiltration and consequently affect tumorigenesis, the study merged the differential gene sets of the seven immune cells and performed pathway enrichment. It can be revealed that most of these gene sets are enriched in pathways related to drug responses and chemical carcinogens, such as P450 cytochrome and other enzymes related to drug metabolism, and receptor activation and DNA synthesis pathways associated with chemical carcinogens. The study suggests that these metabolic pathways may be linked to the differences in tumor immune infiltration and tumorigenesis, providing new perspectives for cancer research. The study presents the specific differential gene sets and pathway enrichment results of the seven immune cell types in the Supplementary Materials, aiming to provide more accurate molecular targets and guidance for clinical cancer treatment (Figure S8).

Finally, to verify the reliability of classification based on immune infiltration fractions, the study performed immune checkpoint evaluations, ESTIMATE scores, and TIDE scores for each subtype. The results of these three scores are shown in the figure (Figure 5C–E). Based on TIDE scores (Figure 5C), it clearly demonstrates that the gene expression of 79 immune checkpoint genes significantly differs between tumor subtypes in most cancers, with fewer differential immune checkpoint genes in THCA compared to other cancers. However, the result reveals that the ESTIMATE and TIDE scores between THCA subtypes show particularly notable differences, compensating for the lack of significant immune checkpoint gene differences in THCA tumors. Therefore, overall, the differences in immune checkpoint evaluations and scores between subtypes of the same tumor type are significant, supporting the accuracy of the classification based on immune cell infiltration fractions. From the perspective of different tumors, the ESTIMATE scores of KIRC subtype 1 and the three LUAD subtypes are significantly higher compared to other tumor subtypes. Additionally, the TIDE scores of ESCA subtype 3 and THCA subtype 3 are significantly higher than those of other subtypes. The trends in immune infiltration fractions between these subtypes are relatively similar, as shown in the increase and decrease table of the seven immune cell infiltration fractions. Therefore, both the differences between subtypes of the same tumor and the similarities between subtypes of different tumors confirm the reliability of the immune infiltration fraction-based classification, thereby supporting the accuracy of the research conclusions.

## 4. Discussion

This study uses DNA methylation genomics for deconvolution to evaluate the immunological basis of the tumor microenvironment in a total of 5323 samples from 14 types of tumors. Based on the immune infiltration of these samples, clustering was performed, revealing significant differences in immune infiltration both between the subtypes and between each category and normal samples. The study identifies commonalities and differences in immune infiltration across the 14 types of tumors and integrates these findings with multi-omics data, such as metabolomics and transcriptomics. As shown in Figure 4A significant differences in survival between subtypes of the four cancer types—KIRC, HNSC, COAD, and PAAD—were revealed in the original study. As shown in Figure 3B and Supplementary Materials Figure S4, the fractions of immune cell infiltration for seven immune cell types in subtypes of 14 cancers were analyzed. For KIRC, HNSC, COAD, and PAAD, significant differences in survival between immune subtypes were observed, and notable differences in CD14+ and CD19+ cell infiltration between subtypes were identified. Based on these observations, this study hypothesizes that the fractions of immune infiltration by CD14+ and CD19+ cells are correlated with patient survival. The result showed that the difference in distribution of CD14+ cells and CD19+ cells may be one of the factors contributing to the different survival outcomes in pan-cancer. Additionally, the differentially expressed genes influencing tumor immune infiltration fractions are primarily enriched in pathways related to drug metabolism and chemical carcinogenesis. After evaluating immune checkpoints and scoring for the 5323 samples, the study found significant differences in the immune scores of the different tumor subtypes identified, further strengthening the reliability of the conclusions. These findings offer directions and recommendations for clinical treatment.

The methylation dataset of the seven immune cells used in this study is consistent with the types of the immune cells datasets selected in previous widely accepted methylation deconvolution studies [21,34], confirming the accuracy and usability of the data in this study. In their study, cell type-specific methylation sites were not identified across all seven cell types. Instead, pairwise differential analyses were conducted between the cell types. In contrast, our study applied the Shannon entropy method to identify methylation sites specific to all seven cell types collectively. These sites were then used as features for cancer subtype classification, representing a different strategy from the previous study. According to conclusions drawn from previous studies [21,35], there are significant differences in immune infiltration fractions and other cells, such as tumor cell infiltration, between samples of a specific type of tumor or tissue. These differences can be used for classification, and such differences are associated with phenotypic characteristics such as survival data [35]. The experimental results obtained in this study also reflect this, further supporting the accuracy of the research findings.

Compared to previous studies, this research has made improvements in two key aspects. Firstly, the QDMR software was used to select cell group-specific methylation gene sites in the study. Compared to previous studies that used methods such as standard deviation for screening, QDMR offers stricter quality control and a more refined selection process, thus improving the methodology for screening specific sites in this study [13,15,29]. Secondly, most previous studies on immune infiltration and tumor cell infiltration focused on a specific type of cancer. In contrast, this study selected 14 types of cancers for research at the pan-cancer level. Unlike previous studies that sought characteristics within a specific cancer type, this study aims to identify general cancer traits and differences between cancers at the pan-cancer level, offering a broader research scope and more comprehensive clinical applicability.

In the future, if DNA methylation data for more cancers and their normal control samples become available beyond the 14 tumors analyzed in this study, new findings in more types of cancers are likely to emerge. Additionally, DNA methylation data from a greater variety of immune cells could provide a broader representation of the characteristics of the tumor microenvironment.

## 5. Conclusions

In summary, this study proposes a method for classifying tumors based on the immune infiltration fractions of seven immune cell types. Significant differences in key characteristics, including gene expression, survival, and two immune scores, were observed across 42 subtypes in 14 tumor types using this method. These findings highlight the heterogeneity of the intra-tumor and inter-tumor immune microenvironment, providing valuable insights for cancer diagnosis and treatment.

## Figures and Tables

**Table 1:** Data distributions in the study.

Database	Tumor/Cell-Types	Number of Samples
TCGA	BRCA	794
	CHOL	45
	COAD	309
	ESCA	186
	HNSC	530
	KIRC	323
	KIRP	276
	LIHC	380
	LUAD	471
	LUSC	370
	PAAD	185
	PRAD	503
	THCA	515
	UCEC	436
GEO	CD4	6
	CD8	6
	CD14	6
	CD19	6
	CD56	6
	Neu	6
	Eos	6

## Data Availability

All authors declare that all data supporting the findings of this study are available in the article. Data and code are available in the following github repository: https://github.com/DAOHUANXIANG/CodeR (accessed on 1 January 2025).

## Supplementary Materials

The following supporting information can be downloaded at: www.mdpi.com/article/10.3390/genes16020160/s1. Figure S1: The results of the KEGG pathway for the cell type-specific genes; Figure S2: The immune infiltration fractions of the 7 immune cells as well as the methylation β value of the 1256 specific sites in 14 tumors; Figure S3: The immune cell infiltration fractions identified tumor subtypes, while t-SNE analysis revealed distinct subtypes in the other 10 tumors; Figure S4: The immune cell infiltration fractions identified tumor subtypes, with a comparison of the immune cell infiltration fractions across the other 10 tumors; Figure S5: The correlation between DNA methylation and gene expression at the 1256 sites across subtypes of the other 10 tumors; Figure S6: The phenotypic characteristics in tumor subtypes, along with survival analysis of the subtypes in the remaining 10 tumors; Figure S7: The phenotypic characteristics in tumor subtypes, with a comparison of phenotypic traits and immune cell infiltration fractions across subtypes in the remaining 10 tumors; Figure S8: The analysis based on transcriptome, presenting the specific differential gene sets and pathway enrichment results for the seven immune cell types; Table S1: The Number of Methylation-Specific Genes Corresponding to Seven Cell Lines; Table S2: The results of the KEGG pathway for the cell type-specific genes; Table S3: Proportions of Immune Cells in Different Clusters Across Various Cancers.
