# Supplementary material for: Evaluation of Pan-Cancer Immune Heterogeneity Based on DNA Methylation
Source: Genes (Basel). 2025 Jan 26;16(2):160. doi: 10.3390/genes16020160 (PMC11855777; doi:10.3390/genes16020160)
Supplement: Supplementary file 1 [file genes-16-00160-s001.zip › Supplementary Figures.pdf]

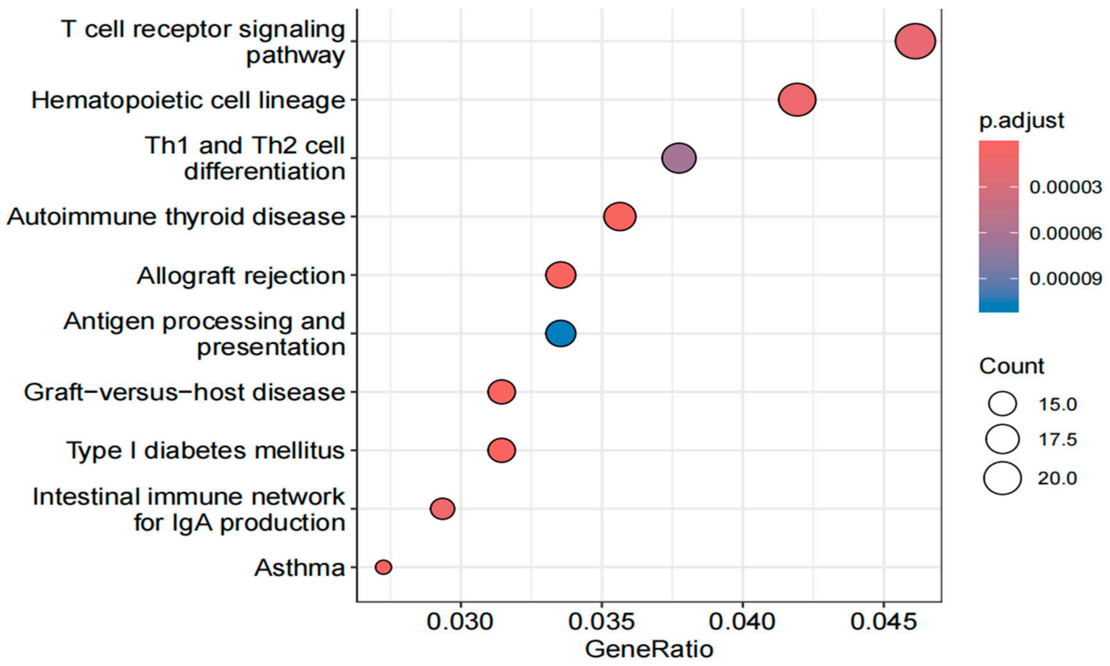

Figure S1. The results of the KEGG pathway for the cell type-specific genes

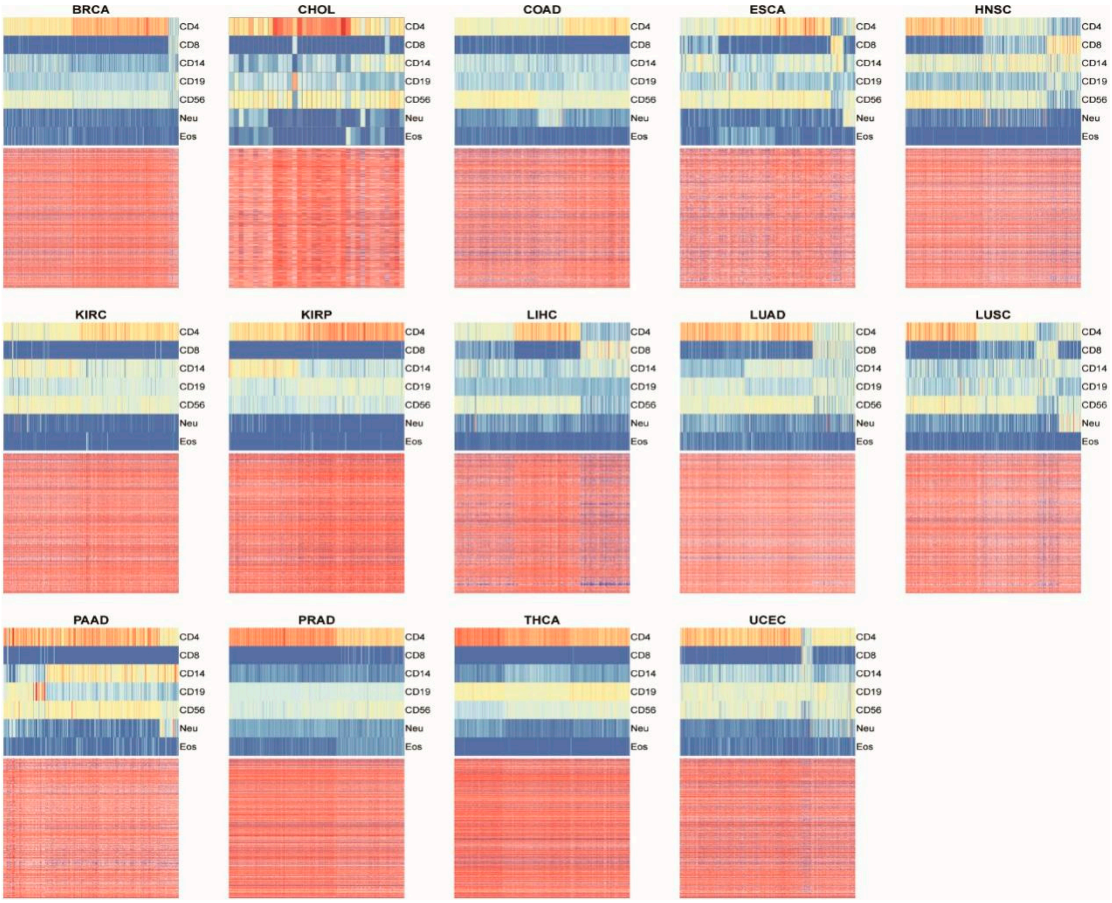

Figure S2. The immune infiltration fractions of the 7 immune cells as well as the methylation beta value of the 1256 specific sites in 14 tumors.

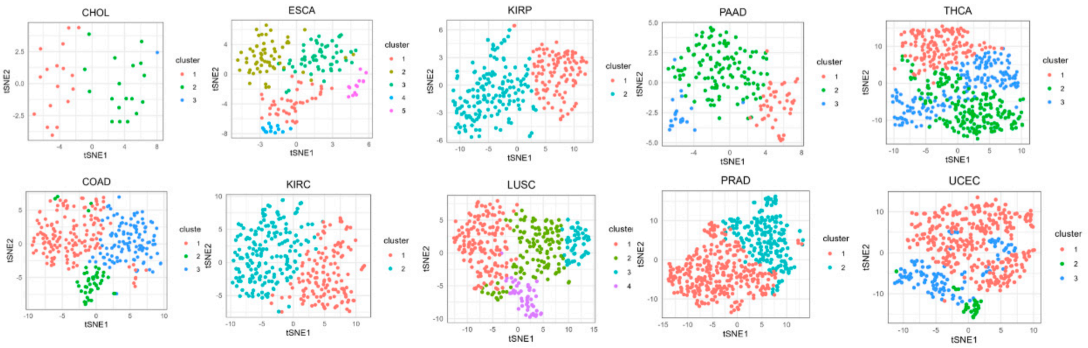

Figure S3. The immune cell infiltration fractions identified tumor subtypes, while t-SNE analysis revealed distinct subtypes in other 10 tumors.

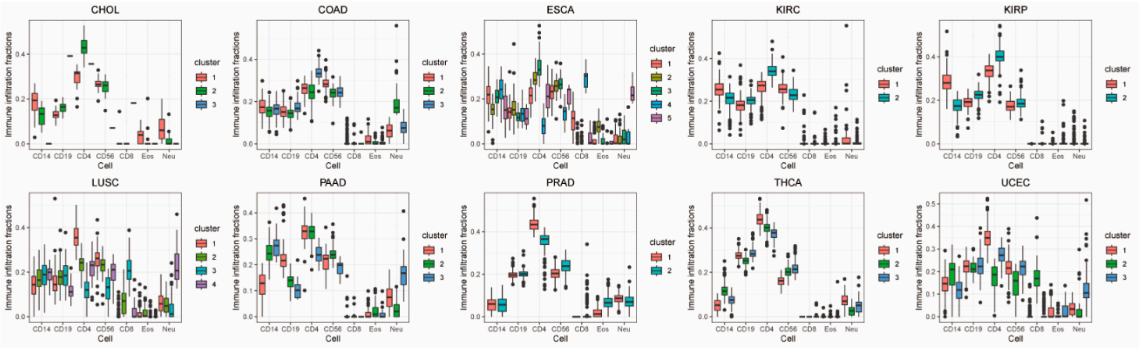

Figure S4. The immune cell infiltration fractions identified tumor subtypes, with a comparison of the immune cell infiltration fractions across other 10 tumors.

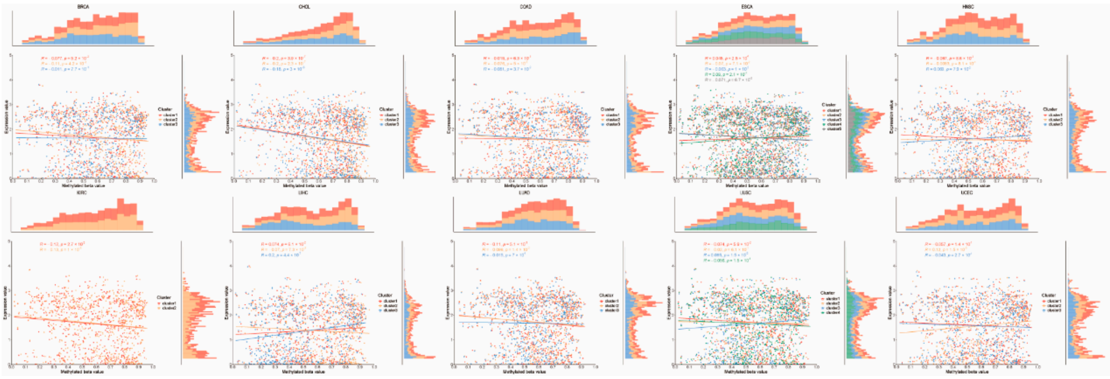

Figure S5. The correlation between DNA methylation and gene expression at the 1256 sites across subtypes of other 10 tumors.

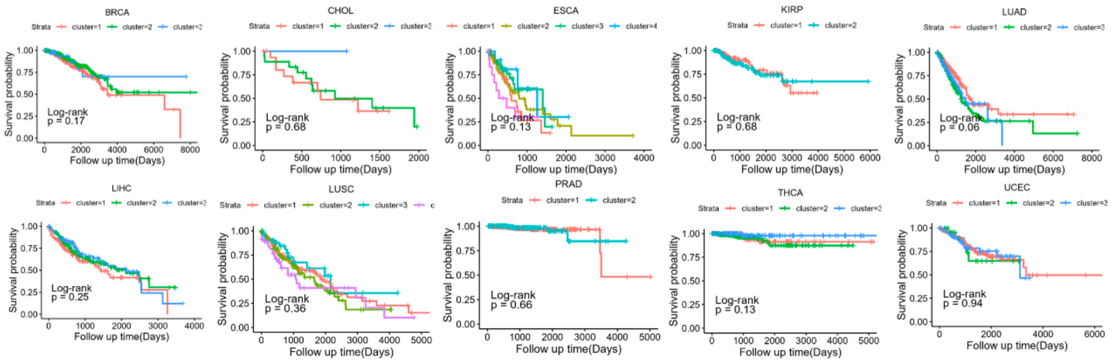

Figure S6. The phenotypic characteristics in tumor subtypes, along with survival analysis of the subtypes in the remaining 10 tumors.

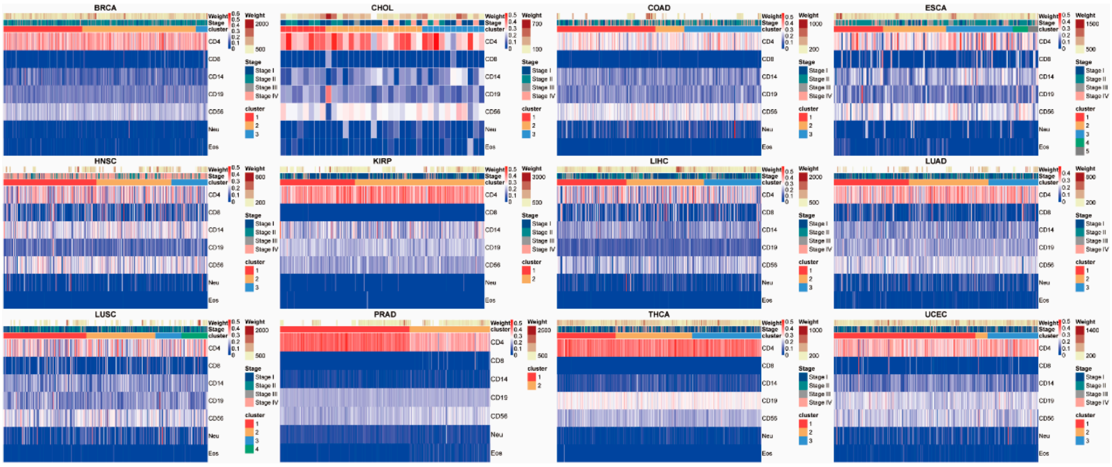

Figure S7. The phenotypic characteristics in tumor subtypes, with a comparison of phenotypic traits and immune cell infiltration fractions across subtypes in the remaining 10 tumors.

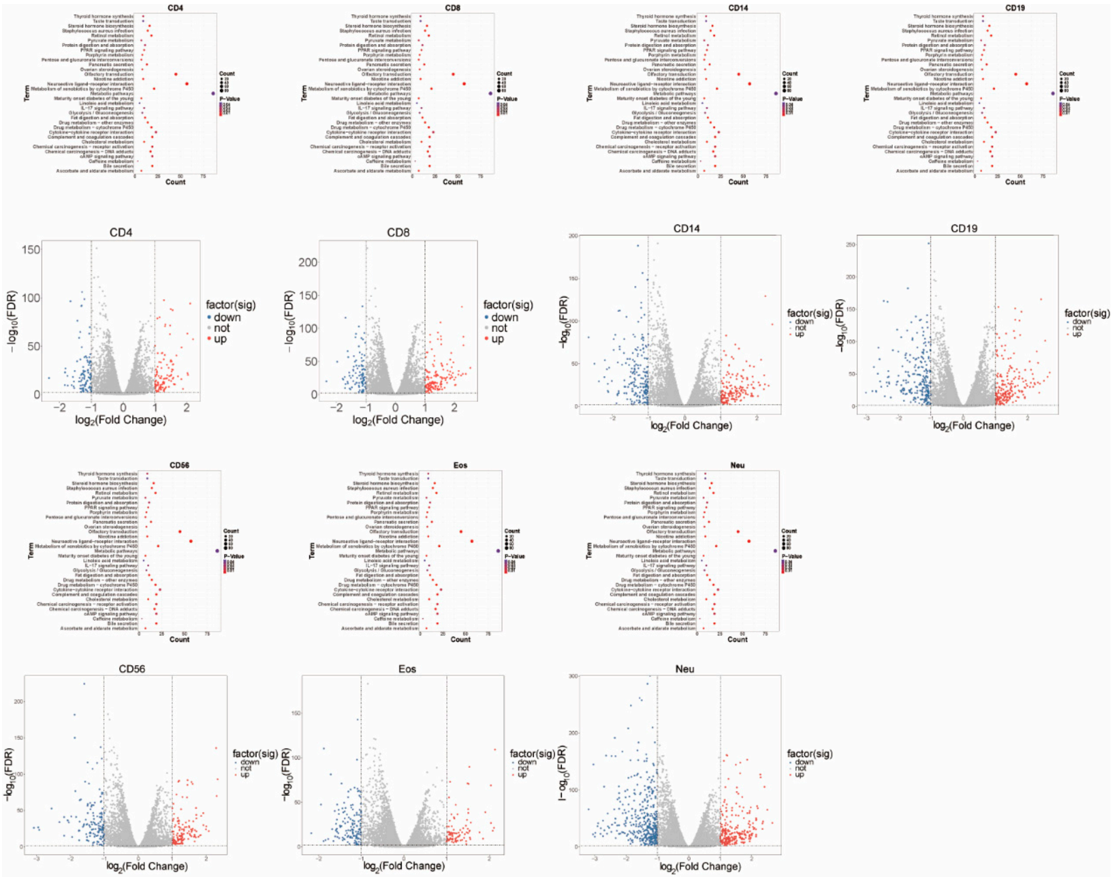

Figure S8. The analysis based on transcriptome, presenting the specific differential gene sets and pathway enrichment results for the seven immune cell type
