# Supplementary material for: Evaluation of Pan-Cancer Immune Heterogeneity Based on DNA Methylation
Source: Genes (Basel). 2025 Jan 26;16(2):160. doi: 10.3390/genes16020160 (PMC11855777; doi:10.3390/genes16020160)
Supplement: Supplementary file 1 [file genes-16-00160-s001.zip › Supplementary Table.pdf]

Table S1. The Number of Methylation-Specific Genes Corresponding to Seven Cell Lines

Cell groups	Number
CD14	149
CD9	294
CD4	194
CD56	86
CD8	190
Eos	289
Neu	54
Total	1256

Table S2. The results of the KEGG pathway for the cell type-specific genes

subcategory	Description	p.adjust
"Immune disease"	Allograft rejection	1. 10E-08
"Immune disease"	Autoimmunethyroid disease	2. 06E-07
"Immune disease"	Graft-versus-host disease	2. 09E-07
"Immune disease"	Asthma	2. 09E-07
"Endocrine and metabolic disease	Type I diabetes mellitus	2. 09E-07
"Immune system"	Intestinal immune network for IgA production	1. 03E-05
"Immune system"	Hematopoietic cell lineage	1. 26E-05
"Immune system"	T cell receptor signaling pathway	1. 94E-05
"Immune system"	Th1 and Th2 cell differentiation	6. 32E-05
"Immune system"	Antigen processing and presentation	0. 000112
"Immune system"	Th17 cell differentiation	0. 000142
"Immune system"	B cell receptor signaling pathway	0. 000152
"Infectious disease: viral"	Epstein-Barr virus infection	0. 000312
"Immune system"	Primary immunodeficiency	0. 000566
"Immune system"	Natural killer cell mediated cytotoxicity	0. 000593
"Cancer:overview"	MicroRNAs in cancer	0. 000599
"Immune system"	Rheumatoid arthritis	0. 000678
"Immune system"	Inflammatory bowel disease	0. 000678

Table S3. Proportions of Immune Cells in Different Clusters Across Various Cancers.("up" represents that the proportion of this cell type in this cluster of the cancer is significantly increased compared to normal tissue. "down" represents that the proportion of this cell type in this cluster of the cancer is significantly decreased compared to normal tissue. "-" represents no significant change.)

tumor	cluster	CD4	CD8	CD14	CD19	CD56	Neu	Eos	Sample Size
BRCA	1	down	up	down	up	up	down	up	306
BRCA	2	up	-	down	up	up	down	up	442
BRCA	3	down	up	down	up	down	down	up	46
CHOL	1	-	-	-	-	-	-	-	17
CHOL	2	up	-	-	up	-	down	-	17
CHOL	3	down	-	up	-	-	down	-	11
COAD	1	down	up	up	down	up	up	down	148
COAD	2	down	up	up	down	up	up	down	44
COAD	3	up	-	up	down	up	up	down	117
ESCA	1	down	up	up	down	up	-	down	41
ESCA	2	-	-	-	-	up	-	down	61
ESCA	3	up	-	up	down	up	-	down	58
ESCA	4	down	up	up	down	down	-	down	13
ESCA	5	down	-	-	down	-	up	down	13
HNSC	1	down	up	up	down	up	down	-	235
HNSC	2	down	up	up	down	-	-	-	192
HNSC	3	down	up	up	-	down	down	-	103
KIRC	1	down	up	up	down	up	down	up	140
KIRC	2	down	up	up	down	up	down	-	183
KIRP	1	down	down	up	down	-	down	-	108
KIRP	2	-	-	up	down	up	down	-	168
LIHC	1	down	up	up	-	-	-	up	129
LIHC	2	up	-	-	-	-	down	up	144
LIHC	3	down	up	up	down	down	down	up	107
LUAD	1	up	up	down	up	-	down	down	173
LUAD	2	-	up	up	up	-	down	down	184
LUAD	3	down	up	down	up	down	down	down	114
LUSC	1	up	up	down	up	up	down	down	151
LUSC	2	down	up	down	up	up	down	down	125
LUSC	3	down	up	down	up	down	down	down	47
LUSC	4	down	up	down	down	-	up	down	47
PAAD	1	-	-	-	-	-	-	-	44
PAAD	2	-	-	up	down	up	down	up	121
PAAD	3	down	down	up	down	down	up	up	20
PRAD	1	up	up	down	-	up	up	-	310
PRAD	2	down	up	down	-	up	up	-	193
THCA	1	up	-	up	down	down	down	-	148

THCA	2	down	down	up	down	up	down	–	192
THCA	3	down	–	up	–	up	down	–	175
UCEC	1	–	–	down	up	down	down	up	302
UCEC	2	down	up	–	up	down	–	–	26
UCEC	3	down	up	down	up	down	up	up	108
